# Lipid metabolism reprogramming in chronic obstructive pulmonary disease

**DOI:** 10.1186/s10020-025-01191-9

**Published:** 2025-04-07

**Authors:** Qianqian Liang, Yide Wang, Zheng Li

**Affiliations:** 1 Fourth Clinical Medical College of Xinjiang Medical University, Urumqi, 830000 Xinjiang China; 2 Xinjiang National Clinical Research Base of Traditional Chinese Medicine, Urumqi, 830000 Xinjiang China; 3 Xinjiang Key Laboratory of Respiratory Disease Research, Urumqi, 830000 Xinjiang China; 4 Xinjiang Clinical Research Center for Respiratory Diseases, Urumqi, 830000 Xinjiang People’s Republic of China

## Abstract

Chronic Obstructive Pulmonary Disease (COPD) is a complex and diverse respiratory disorder, characterized by ongoing respiratory symptoms and restricted airflow. The major clinical manifestations typically encompass chronic cough, sputum production, and wheezing. The main pathological characteristics involve infiltration of inflammatory cells, overproduction of mucus, and damage to the alveolar walls. The underlying causes of COPD are complex and remain incompletely elucidated, thought to originate from the combined effect of various factors. Lipids, as hydrophobic molecules, fulfill three fundamental functions: energy storage, membrane biosynthesis, and signal transduction. Lipid metabolism is intricately intertwined with various metabolic pathways and plays a pivotal role in the complex pathogenesis of COPD. Delving into lipid metabolism, as well as the particular modifications and roles of lipid molecules in cells, is of paramount importance in the context of COPD. This review primarily aims to elucidate the role of fatty acid metabolism in the onset and progression of COPD. Additionally, it examines the potential of lipid metabolism reprogramming as a promising therapeutic approach, illuminating new paths for the management and treatment of this disabling respiratory condition.

## Introduction

**Fig. 1:**
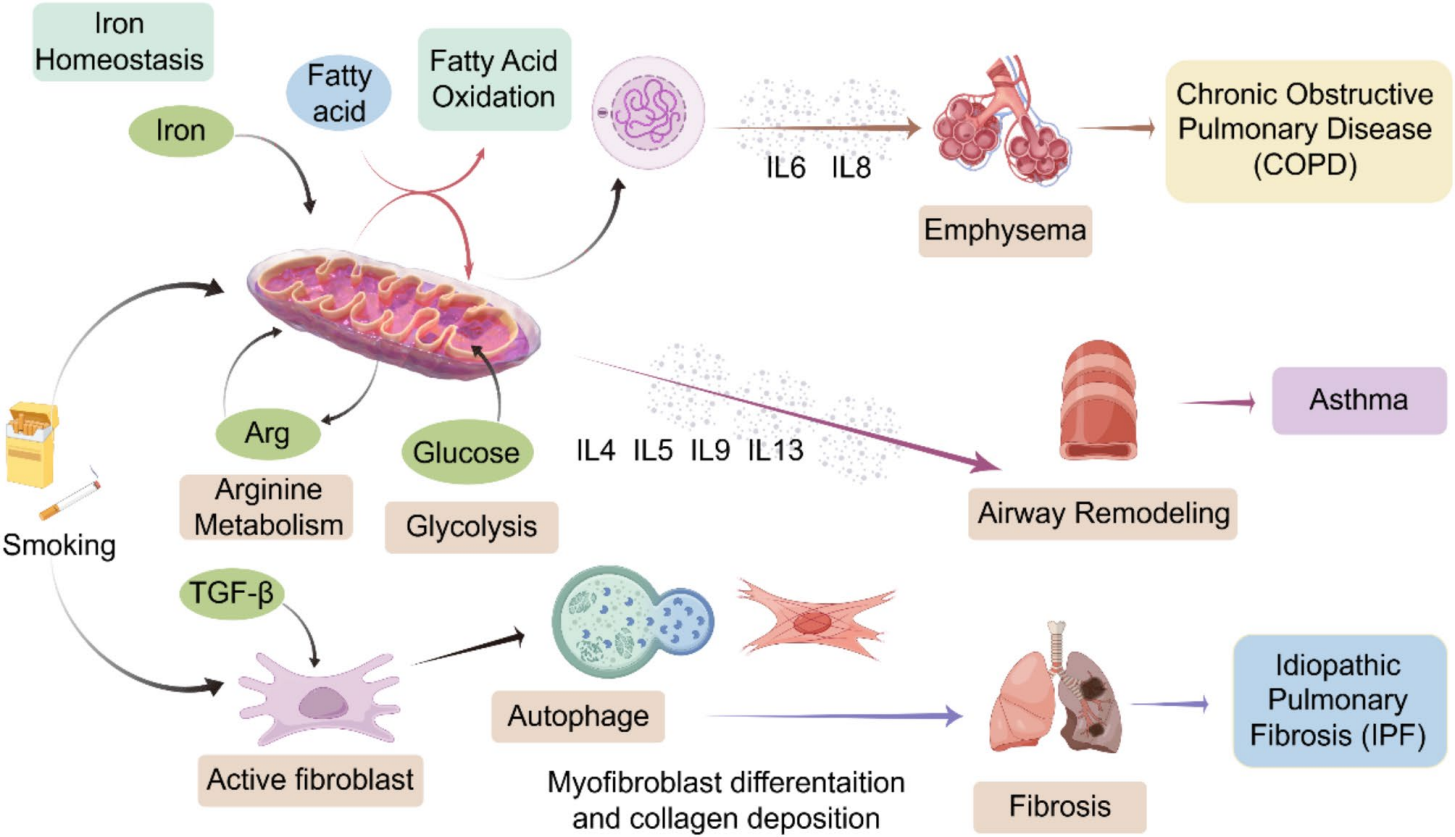
A promoter of cigarette smoke-elicited inflammatory lung ailments. It is extensively recognized as a significant risk factor for diverse lung diseases, including COPD, asthma, and pulmonary fibrosis. Metabolomics and its techniques for metabolic reprogramming have made significant advancements in understanding and treating lung diseases

Chronic obstructive pulmonary disease (COPD) is a varied respiratory ailment defined by constant respiratory issues and limited air passage. Main clinical manifestations include chronic cough, sputum production, and wheezing, with primary pathological features encompassing inflammatory cell infiltration, excessive mucus secretion, and destruction of alveolar walls (Chen et al. 2019). This chronic respiratory ailment results from progressive airflow obstruction attributed to abnormalities in the airways and/or alveoli, comprising chronic bronchitis, airway remodeling, and emphysema, all contributing to impaired lung function. COPD poses significant economic and health burdens on society (Liu and Summer 2019; Zhang et al. 2018). An increasing amount of studies suggests that disturbances in cellular metabolism are closely intertwined with the development of numerous diseases, including COPD, which is often accompanied by bodily concurrent illnesses such as cardiac ailments, metabolic issues, and bronchiectasis (Jiang et al. 2023). The etiology of COPD is intricate and not fully elucidated, currently understood as the result of multiple factors acting in concert (Zhang et al. 2018; Jin and Yuan 2020). Primarily, chronic irritants like tobacco smoke affect the lungs, triggering abnormal inflammatory responses in the airways, lung tissue, and blood vessels of the lungs (Fig. 1). This leads to chronic inflammation characterized by infiltration of neutrophils, macrophages, and lymphocytes, which release a wide array of inflammatory mediators that act on structural cells in the airways and lung tissue (Barnes 2016). This cascade further stimulates the aggregation of T lymphocytes (particularly CD8+), eosinophils, and neutrophils in lung tissues, leading to the emission of various mediators including interleukin-8 (IL-8), leukotriene B4 (LT-B4), tumor necrosis factor-α (TNF-α), interleukin-17 (IL-17), interleukin-6 (IL-6), causing structural damage to lung tissues (Fedele et al. 2024). Autonomic nervous system dysregulation, increased cholinergic nerve tone, oxidative-antioxidant imbalance, and protease-antiprotease imbalance exacerbate lung inflammation and airflow limitation in COPD patients (Fedele et al. 2024). Hereditary elements also play a part in the progression of the disease. The disease lacks typical early symptoms and signs, making diagnosis challenging and resulting in suboptimal awareness, diagnostic rates, and treatment rates (Z)hou et al. 2025).

The determination of COPD relies on a comprehensive analysis of patient risk factors, clinical symptoms, signs, and lung function. The essential criterion for diagnosis is partially reversible airflow limitation, with confirmation often based on forced expiratory volume in 1 s (FEV1) / forced vital capacity (FVC) < 70% after bronchodilator use. In some cases, patients may lack significant symptoms like cough, sputum production, or dyspnea, and COPD may be diagnosed solely based on detecting FEV1/FVC < 70% during lung function tests, after excluding other conditions (Kotlyarov and Bulgakov 2021). An FEV1/FVC ratio less than 0.7 is the gold standard for COPD diagnosis but cannot differentiate between different COPD subtypes. Studies have revealed abnormal levels of lipids, amino acids, carbohydrates, and nucleotide metabolism in COPD patients, playing crucial roles in disease progression (Xu et al. 2021).

Lipids, as hydrophobic molecules, fulfill three fundamental functions: energy conservation, membrane formation, and signal relay. Lipid metabolism, which includes anabolism and catabolism, is divided into categories like triglycerides, cholesterol, phospholipids, and glycolipids. It involves synthesizing new lipids from smaller molecules and oxidizing lipids to produce energy or other lipid mediators (Barnes 2016). This metabolic realm is intricately associated with diabetes, cancer, neurodegenerative ailments, and cardiovascular conditions. Previously viewed as static reservoirs of bioenergetics, lipids have come to the fore as vital components of intracellular signaling routes, with significant clinical ramifications in modulating host inflammatory responses (Hsieh et al. 2023). Ongoing research is unveiling novel lipid mediators governing inflammation dynamics. Interconnected with multiple metabolic pathways, lipid metabolism plays pivotal roles in the intricate etiology of COPD (Bowerman et al. 2020).

The lung, a sophisticated organ made up of various cell types, witnesses distinct regulation of lipid molecules across various cells in COPD, potentially exerting differential impacts on the ultimate pathogenesis of the disease. Hence, investigating lipid metabolism and the particular modifications and roles of lipid molecules within cells in the context of chronic obstructive pulmonary disease assumes paramount importance (Huang et al. 2024; Liu et al. 2022a, b). This review focuses primarily on elucidating the function of fatty acid metabolism in the development and advancement of COPD, while also delving into the potential of lipid metabolism reprogramming as a promising therapeutic modality.

## The important role of lipid metabolism in COPD

Malnutrition is an independent and reversible significant prognostic indicator in COPD patients. Nutrient metabolism primarily involves carbohydrate, lipid, and protein metabolism. Malnutrition is primarily characterized by cachexia. Research indicates that cachexia in COPD patients is mainly linked to skeletal muscle dysfunction and has a significant correlation with lipid metabolism. Recent research has indicated that individuals with COPD display irregularities in lipid metabolism, and lipid metabolism abnormalities are one of the main contributors to atherosclerosis development (Zhao et al. 2020). Atherosclerosis can lead to cardiovascular complications, increasing the incidence of cardiovascular diseases in patients and influencing the progression and prognosis of COPD (Boots et al. 2003).

COPD patients often present with abnormalities in lipid metabolism, with differences observed in lipid parameters such as triglyceride (TG), total cholesterol (TC), high-density lipoprotein cholesterol (HDL-C), low-density lipoprotein cholesterol (LDL-C), apolipoprotein A (APO A), and apolipoprotein B (APO B) often show discrepancies in COPD patients relative to healthy individuals compared to healthy individuals. However, studies have shown variations among these parameters (Wang et al. 2024). As the disease progresses, lipid levels tend to decrease, but the degree of decline varies among individuals due to factors such as individual differences and geographical variations (Wang et al. 2023a, b, c). The decrease in lipid levels can be influenced by factors such as malnutrition, which, when prolonged, can lead to a reduction in subcutaneous fat and the gradual onset of cachexia in COPD patients. The changes in blood lipid levels reflect the systemic lipid metabolism status to a certain extent (Calvier et al. 2022). Moreover, abnormalities in lipid metabolism are observed in COPD patients both during stable and exacerbation periods, reflecting lipid metabolic abnormalities. APO A1, a major apolipoprotein in HDL, is vital for preserving the structure of HDL and in plasma lipidation and transport. A significant decrease in APO A1 levels can accelerate atherosclerosis formation (Fan et al. 2023). The decreased levels of high-density lipoprotein in COPD patients significantly reduce the clearance rate of cholesterol in blood vessels, increasing the likelihood of atherosclerosis development. In the mid to late stages of COPD, the lipid levels in the serum are significantly reduced, with multiple factors contributing to this condition, with hypoxemia being the primary factor. Abnormal levels of apolipoproteins may better reflect lipid metabolic abnormalities compared to abnormalities in other components. The significance of lipid metabolism in COPD has attracted notice.

### Fatty acid metabolism and metabolic reprogramming in COPD

**Fig. 2:**
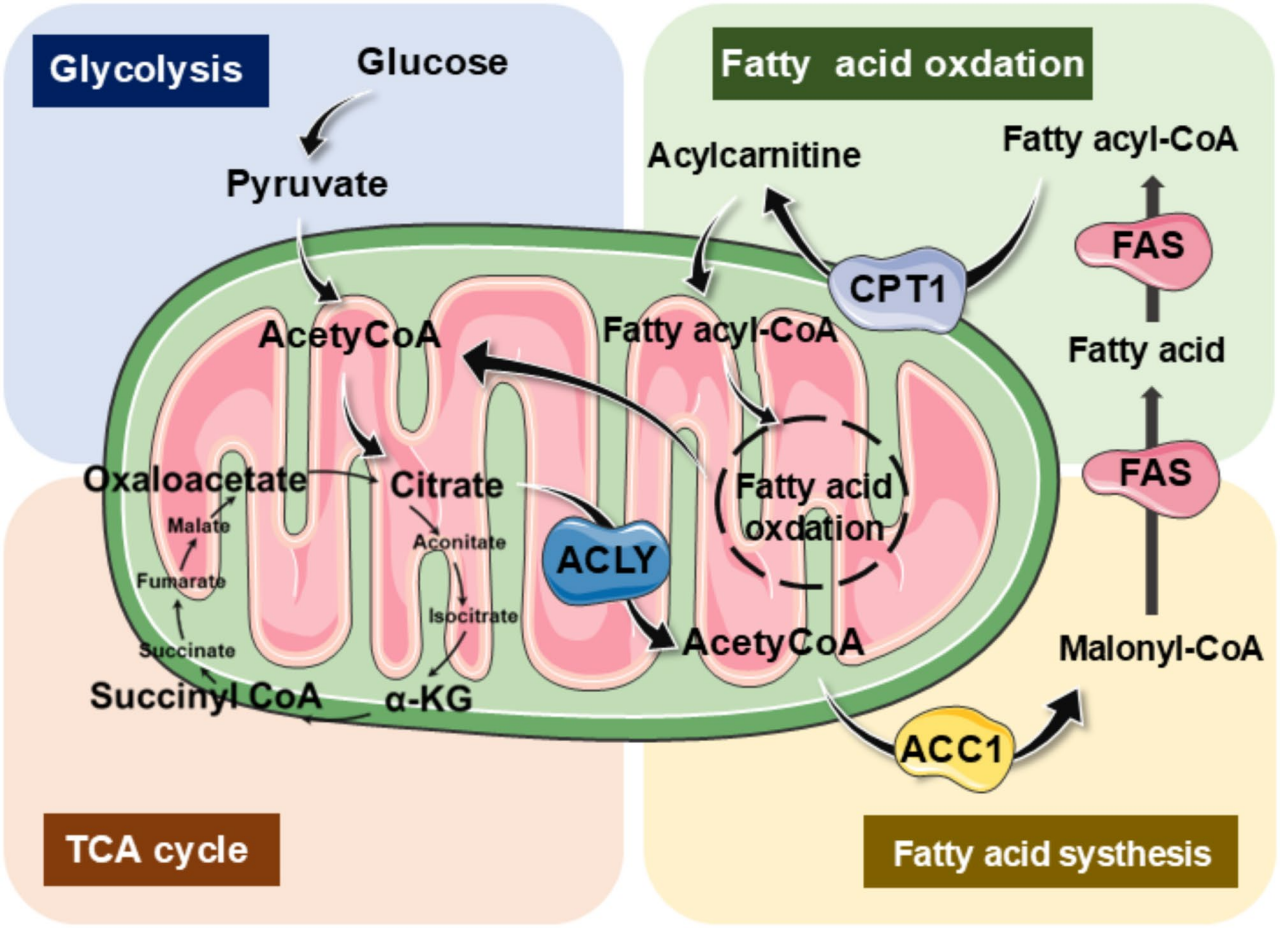
Illustrations of metabolic pathways including glycolysis, FAS, and FAO. The process of glycolysis transforms glucose into pyruvate, which has the option to join the TCA cycle. Additionally, glycolysis provides materials for the pentose phosphate pathway (PPP), responsible for producing ribose needed for nucleotides and NADPH. Citrate can either be completely oxidized to produce ATP or be moved to the cytoplasm, where it is converted back to acetyl-CoA by ATP citrate lyase (ACLY). A portion of acetyl-CoA is altered by acetyl-CoA carboxylase 1 (ACC1) to create malonyl-CoA. Fatty acid synthase (FAS) combines acetyl-CoA and malonyl-CoA to synthesize the 16-carbon saturated fatty acid palmitate along with other saturated long-chain fatty acids. In the cytoplasm, acyl-CoA synthetase (ACS) activates fatty acids by turning them into acyl-CoA. At the outer mitochondrial membrane, carnitine palmitoyltransferase 1 (CPT1) transforms acyl-CoA into acylcarnitine, which is subsequently conveyed to the mitochondrial matrix. Upon reaching the mitochondrial matrix, acyl-CoA is subjected to FAO, converting it into acetyl-CoA

Fatty acids constitute a significant portion of lipids and are vital for energy metabolism. Fatty acid oxidation (FAO) serves as a key energy - producing process, which is enhanced during extended fasting, physical activity, or metabolic strain (Fig. 2). Fatty acids are unable to permeate mitochondrial membranes. as a result, they need to bind to carnitine for mitochondrial entry. Carnitine, facilitated by carnitine palmitoyltransferase 1 (CPT1) on the inner mitochondrial membrane, creates a high-energy ester linkage with fatty acids, generating acylcarnitine. The carnitine-acylcarnitine translocase (CACT) facilitates the exchange of acylcarnitine and carnitine across the external and internal membranes of the mitochondria, eventually converting acylcarnitine back to acyl-CoA to undergo oxidation via CPT2 (Yoshida et al. 2019). Fatty acids serve as a crucial energy supply, since FAO generates 2.5 times the amount of ATP per mole compared to glucose oxidation. Although a range of factors, such as genetic predispositions or environmental exposures, may play a role in the progression of COPD, smoking is still the predominant risk factor. Subchronic inhaling cigarette smoke (8 weeks) leads to decreased glucose metabolism in alveolar cells. This damage is offset by increased FAO to preserve cellular energy balance, along with a rise in CPT1 expression. Increased utilization of palmitic acid (likely derived from di-palmitoyl phosphatidylcholine) has been observed in type II alveolar cells. Consistently, exposure to cigarette smoke promotes FAO and enhances respiratory function of mitochondria in human bronchial epithelial cells (Zhu et al. 2024).

Viral infections can trigger acute exacerbations in COPD patients. Research indicates that the activity of fatty acid synthesis is enhanced when bronchial epithelial cells are infected with rhinovirus, and inhibiting fatty acid synthesis reduces rhinoviral infections (Kazeminasab et al. 2020). Therefore, fatty acid synthesis may represent a candidate for exacerbations in COPD. Extended exposure to tobacco smoke may result in a range of metabolic alterations (Wang et al. 2023a, b, c). The Warburg effect, which is characterized by tumor cells consuming glucose at a high rate and released the majority of glucose-derived carbon as lactate instead of completely oxidizing it, may contribute to the development of inflammation (Agudelo et al. 2020a, b). Likewise, in human bronchial epithelial BEAS2B cells exposed to cigarette smoke condensate for 7 months, glucose uptake and lactate generation were enhanced (Kotlyarov 2021). Moreover, the ability to synthesize lipids and the process of net reductive carboxylation were both increased, indicating significant metabolic reprogramming in the airway epithelium. While it is still a hypothesis, alterations in lipid metabolism could potentially account for the increased susceptibility of COPD patients to lung cancer (Lundström et al. 2011). Moreover, lipid metabolism abnormalities play a crucial role in both obese and low-BMI COPD patients. In obese individuals, dysfunctional adipose tissue leads to disrupted lipid metabolism, increased secretion of pro-inflammatory factors, and exacerbation of COPD inflammation. On the other hand, low-BMI patients, due to reduced adipose tissue volume and diminished lipid metabolism capacity, are unable to effectively regulate inflammation, resulting in persistent inflammatory responses (Kotlyarov 2021). Additionally, both obese and low-BMI patients exhibit abnormal levels of leptin and adiponectin, adipokines that further impact the course of COPD by modulating inflammation and immune responses. Obesity and low BMI significantly influence the progression of COPD through their effects on lipid metabolism and inflammatory responses. Despite the heavier inflammatory burden in obese patients, their relatively better nutritional reserves contribute to a comparatively favorable prognosis (Lahousse et al. 2013). In contrast, low-BMI patients experience poorer outcomes due to malnutrition and decreased lipid metabolism capacity, leading to challenges in controlling inflammation. Therefore, in the clinical management of COPD, attention should be given to the nutritional status and lipid metabolism of patients. Tailored nutritional interventions and inflammation management strategies should be implemented to improve patient outcomes (Kazeminasab et al. 2020).

### Characteristics of disrupted fatty acid metabolism in COPD

Although a multitude of elements, such as genetic and environmental aspects, can play a part in the onset of COPD, smoking stands out as the most significant risk factor. Following a certain duration and concentration of tobacco exposure, the levels of FAO and oxidative phosphorylation enzymes in lung cells undergo alterations. Mitochondrial metabolism enhances to adapt to smoke exposure. Short-term tobacco exposure can lead to mitochondrial fragmentation and increased mitochondrial reactive oxygen species. After 8 weeks of tobacco exposure, glycolysis decreases in AEC2 cells, compensating to maintain ATP production, with increased FAO and upregulation of the CPT system. After 12 months of tobacco exposure, to meet the increased oxygen demand, the expression of proteins related to FAO increases, as does the antioxidant enzyme content (Lahousse et al. 2013). Nevertheless, COPD patients exhibit a reduced or disrupted rate of fatty acid metabolism in their lung tissue. This suggests that FAO could serve a double purpose in the development of COPD, initially supporting mitochondrial metabolic demands in the short term but resulting in higher mitochondrial reactive oxygen species (ROS) production and greater oxidative stress risk associated with long-term tobacco exposure. Additionally, lipid metabolism decreases as the severity of the disease progresses (Zhao et al. 2023).

On the other hand, enhanced FAO can reduce lipotoxicity, or by activating long-chain fatty acids, peroxisome proliferator-activated receptors (PPARs), and stimulating CPT1 activity to decrease free fatty acids, thereby providing organ protection (Baralla et al. 2018). PPARγ, which belongs to the PPAR transcription factor subfamily, governs glucose and lipid metabolism, inflammation, and cell proliferation. In COPD patients, lung tissues, epithelial cells, and marrow cells display reduced PPARγ expression (Wang et al. 2023a, b, c). Various oxidative stress-related metabolites, such as choline, isopropanol, and nitroso carnitine, were detected in induced sputum of COPD patients, and the superoxide dismutase of severe COPD patients was significantly higher than that of moderate COPD patients.

Enhanced FAO in the early stages of COPD is a compensatory reaction to elevated oxidative stress byproducts, and may be partially compensated by related protective regulations. However, in the later stages of COPD, the reduced FAO and diminished protective regulations, coupled with the upregulation of oxidases, lead to the production and inadequate clearance of free fatty acids, oxidative stress products, and toxic lipid compounds, resulting in cellular tissue damage (Viglino et al. 2017). Owing to the intricate connection during fatty acid metabolism pathways and oxidative stress responses, and the challenges in balancing the effectiveness and precision of antioxidant medications, further research into the specific mechanisms of lipid metabolism at different time points in COPD is needed to provide targets for alleviation and treatment (He et al. 2023; Singh et al. 2019).

Lipid metabolism is vital for the metabolic reprogramming of all immune cell populations in COPD lung tissues (Takeda et al. 2015). Smoke entering the lungs activates Toll-like receptor 4 (TLR4) in membrane lipid rafts, triggering the activation of innate immune cells, structural cells, and damage-associated molecular patterns, resulting in the secretion of various cytokines, chemokines, lipid mediators, and more (Minagawa et al. 2020). Activated dendritic cells induce T and B cell responses, forming lymphoid follicles and mediating persistent chronic inflammatory reactions (Takeda et al. 2015). Metabolic shifts in fatty acid metabolism among healthy smokers promote inflammation, with increased levels of interleukin-5 (IL-5), interleukin-9 (IL-9), and interferon-alpha. The sustained activation of the NLR family pyrin domain-containing protein 3 (NLRP3) inflammasome is associated with innate immune abnormalities in COPD. CPT1a oxidizes palmitate, activating NLRP3 inflammasomes to promote reactive oxygen species production. In other inflammatory diseases like sepsis, CPT1a can stimulate macrophage lipid synthesis and activate NLRP3 through mitochondrial uncoupling protein 2.

CD28 serves as the initial signal for T cell activation, increasing the expression of CPT1a to enhance mitochondrial metabolic capacity, utilizing FAO to maintain mitochondrial cristae density and reserve respiratory capacity. Activation of the mammalian target of rapamycin (mTOR) signaling pathway can mediate inflammatory responses, induce cellular senescence, and differentiate CD4 + T cells into effector T cells. Imbalances in the inflammatory regulation between Th17 and Treg cells can reflect the progression of COPD with comorbid type 2 diabetes (Takeda et al. 2015).

Traditional macrophages are categorized into pro-inflammatory M1 macrophages and anti-inflammatory M2 macrophages depending on their activation state. The activation of M1 macrophages causes metabolite accumulation, such as succinate accumulation, which can stimulate chronic inflammation through the hypoxia-inducible factor and interleukin-1β, with metabolic changes showing increased fatty acid synthesis, decreased FAO, and mitochondrial fission (Barnes 2004). Conversely, the activation of M2 macrophages leads to increased FAO, higher efficiency of mitochondrial oxidative phosphorylation, and potential transformation into M1 macrophages (Cakmak et al. 2023). Macrophages can also be categorized based on their tissue location, dividing into alveolar and interstitial types, with their proliferation and activation influenced by the unique lung environment (Cakmak et al. 2023). External factors such as cigarette smoke can induce oxidative stress, increase ROS levels, and consequently impact macrophage polarization. The elevation of ROS levels can promote macrophages towards M1 polarization. For instance, iron ions induce ROS production, enhance p53 acetylation, thereby driving macrophages towards M1 polarization. Furthermore, M1 macrophages exhibit significant metabolic reprogramming during activation, including enhanced glycolysis and alterations in the TCA cycle. These metabolic changes further stimulate ROS production, establishing a positive feedback loop that exacerbates the inflammatory response. In contrast, the metabolic characteristics of M2 macrophages are opposite to M1, with their metabolic pathways favoring aerobic oxidation and producing fewer ROS. In COPD, oxidative stress-induced ROS not only affects macrophage polarization but may also impair mitochondrial function, leading to cellular senescence and phagocytic dysfunction. This metabolic imbalance and abnormal macrophage function collectively contribute to the progression of inflammation and tissue damage in COPD. Therefore, modulating the metabolic state and polarization direction of macrophages could potentially serve as a therapeutic strategy for COPD treatment (Cakmak et al. 2023).

Lipid metabolism can trigger inflammatory responses through lipid-metabolizing enzymes, activation of signaling pathways, or induction of oxidative stress, leading to innate immune reactions. On the other hand, some lipid-related substances also possess anti-inflammatory properties. Nevertheless, additional studies are required to comprehend the precise changes and interplay between the pro-inflammatory and anti-inflammatory impacts induced by lipid metabolism in COPD (Santus et al. 2005).

### Degradation of lipid mediators in COPD

Eicosanoids constitute a powerful group of bioactive signaling molecules, functioning as local messengers that affect tissues near their site of production. In inflammatory responses, phospholipase A2 acts on cell membrane phospholipids, freeing arachidonic acid (AA) from the central carbon of glycerol. Arachidonic acid, a 20-carbon fatty acid containing four double bonds, is a controlled substrate for phospholipase A2 hydrolysis and a precursor to numerous lipid signal transducers. Cyclooxygenases (COX) 1/2 catalyze the oxidation of AA, generating the prostaglandin family of oxidized fatty acids. Generally, these lipid substances can exhibit pro-inflammatory properties, spreading their signaling impacts via receptor-mediated pathways and adding to various inflammatory events (Chung 2005). Prostaglandins, derived from arachidonic acid, constitute a family of metabolites including PGD2, PGE2, PGF2, PGI2, and thromboxane A. Increased concentrations of PGE2 have been observed in the respiratory secretions of COPD patients, potentially fostering airway inflammation and compromised lung repair (Hunninghake 2005).

Leukotrienes, which are another set of inflammatory mediators produced inside cells from arachidonic acid by 5-lipoxygenase, are generated by activated macrophages, neutrophils, and epithelial cells in COPD. Cysteinyl leukotrienes (LTC4, LTD4, LTE4) and Leukotriene B4 (LTB4) are included among these compounds. LTB4, a potent inflammatory mediator and chemoattractant for neutrophils and T-cells, shows increased concentrations in induced sputum during exacerbations, indicating its role in exacerbating inflammation (Jin et al. 2023).

Moreover, nitrated fatty acids (NFAs), which are produced within the body through reactions between nitrogen oxide-derived species and unsaturated fatty acids, possess notable anti-inflammatory properties. Treatment with NFAs lowers the expression and activity of the inflammatory transcription factor NF-κB while increasing PPAR-γ levels. Additionally, it reduces the generation of inflammatory cytokines, chemokines, and the protease cathepsin S (Cat S), a key factor in alveolar damage associated with emphysema (Li et al. 2020).

In recent years, research has revealed that pro-resolving lipid mediators, including lipoxins, resolvins, maresins, and protectins, play a vital role in regulating inflammation and disease progression in chronic obstructive pulmonary disease (COPD). These lipid mediators are endogenous anti-inflammatory agents capable of promoting the resolution of inflammation, playing a crucial role in the pathophysiological processes of COPD. Lipoxins are metabolites of arachidonic acid synthesized by various cells such as monocytes, macrophages, respiratory epithelial cells, and vascular endothelial cells. They exhibit significant anti-inflammatory properties, inhibiting the movement of inflammatory cells and the generation of inflammatory mediators, thus facilitating the resolution of inflammation (Cakmak et al. 2023). Resolvins, another important group of pro-resolving lipid mediators, mainly originate from polyunsaturated fatty acids such as omega-3 fatty acids. They regulate the inflammatory response, promote the clearance of inflammatory cells, and aid in tissue repair. Decreased levels of resolvins in COPD patients may be associated with sustained inflammation. Maresins primarily function during the resolution phase of inflammation. By modulating the activity of inflammatory cells and promoting tissue repair, they help alleviate inflammation. While the mechanisms of action of maresins in COPD require further investigation, their anti-inflammatory properties make them a potential therapeutic target. Protectins, another class of lipid mediators derived from omega-3 fatty acids, possess anti-inflammatory and neuroprotective effects (Li et al. 2020). They play a crucial role in regulating the inflammatory response and promoting tissue repair. Changes in the levels of protectins in COPD may be related to persistent inflammation and tissue damage. Modulating the levels of these lipid mediators may help control the inflammatory response and alleviate symptoms in COPD patients.

The progression of COPD typically accelerates with exacerbations. The primary treatment goal intended to decrease the frequency of exacerbations (Dailah 2022). Hence, there is a need for specific biomarkers to monitor and diagnose exacerbations. Considering the roles of these lipid mediators in COPD, further research is necessary to explore their potential as biomarkers for exacerbations (Drozdovszky et al. 2014). Clearly, lipid mediators are vital in regulating the start and end of inflammation by maintaining a balance between pro-inflammatory and anti-inflammatory substances. Nonetheless, the mechanism by which the lungs transition from secreting pro-inflammatory molecules to anti-inflammatory molecules remains enigmatic (Jiang et al. 2017; Liu et al. 2022a, b). Additional research is required to directly assess the effect of lipid metabolism genes on the progression of COPD, which is essential for generating various lipid mediators.

## Molecular mechanism of lipid metabolic reprogramming

### Normal fatty acid metabolic pathway

Fatty acids, being a primary constituent of lipids, are pivotal in a fundamental biological energy pathway. Long-chain fatty acids, ranging from 13 to 21 carbons, are triggered by sodium-dependent acyl-CoA synthetase 1 on the outer mitochondrial membrane, forming acylcarnitine via the crucial enzyme CPT1. This acylcarnitine is transported into the mitochondrial matrix via the carnitine-acylcarnitine translocase, and then it gets transformed back into acyl-CoA by CPT2 on the matrix side (Kotlyarov 2022). Subsequently, through multiple cycles of dehydrogenation, hydration, dehydrogenation, and thiolysis via the fatty acid beta-oxidation enzyme series, acetyl-CoA, flavin adenine dinucleotide, and nicotinamide adenine dinucleotide are produced (Pniewska and Pawliczak 2013; Nambiar et al. 2020). Carnitine is then transported back to the mitochondrial intermembrane space via organic cation transporter 2, maintaining carnitine homeostasis and ensuring the normal operation of the mitochondrial fatty acid beta-oxidation system (Arezina et al. 2023).

Fatty acid synthesis (FAS) involves a series of steps facilitated by acetyl-CoA carboxylase 1, initiation of the FAS complex, loading of two-carbon units, condensation, reduction, and re-reduction. The initial synthesis product, acyl carrier protein (ACP), cycles multiple times to ultimately generate palmitate, phospholipids, triglycerides, and fatty acids of varying carbon chain lengths (Agudelo et al. 2020a, b). This process plays a crucial role in energy storage, membrane formation, and signal propagation, utilizing products from various other metabolic pathways, particularly glycolysis, the tricarboxylic acid cycle, and the PPP (Rahman 2005). Glycolysis powers the PPP, which produces ribose for nucleotides and NADPH. Citrate generated in the tricarboxylic acid cycle can be transported from the mitochondria to the cytosol through the citrate carrier and is then converted into acetyl-CoA by ACLY (Gao et al. 2024). Fatty acid synthesis occurs in the cytoplasm, starting with the carboxylation of acetyl-CoA to malonyl-CoA, a process that requires ATP. This essential step in fatty acid synthesis is facilitated by ACC1, followed by FAS, a pivotal multifunctional enzyme that, with NADPH as a co-factor, combines acetyl-CoA and malonyl-CoA to produce saturated 16-carbon fatty acid palmitate and other saturated LCFAs (Biswas et al. 2013). Saturated LCFAs may be further modified by elongases or desaturases to generate more complex lipids, subsequently employed in the production of phospholipids, triglycerides, and cholesterol esters (Yang et al. 2024).

AMP-activated protein kinase (AMPK) phosphorylates acetyl-CoA carboxylase 1 to deactivate it. Persistent activation of AMPK reprograms cellular metabolism, favoring FAO as an energy source by limiting glucose and fatty acid synthesis (Mizumura and Gon 2021). Cholesterol synthesis is regulated by hydroxymethylglutaryl-CoA reductase in the cytoplasmic endoplasmic reticulum, exhibiting a circadian rhythm and being modulated by cholesterol, sterol regulatory element-binding proteins (SREBPs), cholesterol-induced multi-nucleation, proteasomal degradation, and AMPK phosphorylation (Catteau et al. 2021).

FAO communicates with glucose metabolism through the tricarboxylic acid cycle. When glycolysis is active, high concentrations of citrate in the mitochondria and cytosol promote the conversion of acetyl-CoA carboxylase to an active polymer, facilitating fatty acid synthesis. Succinate accumulation inhibits CPT1 by elevating levels of acyl-CoA dehydrogenase, suppressing FAO (Yu et al. 2021). Flavin adenine dinucleotide enters the electron transport chain complex III, while nicotinamide adenine dinucleotide enters complex I, producing ATP. When electrons reach complex IV, oxygen accepts the electrons, reducing to superoxide anions, peroxide radicals, and binding closely with complex IV (Sicinska et al. 2017). Under abnormal conditions, hydrogen peroxide and hydroxyl radicals are generated, acting as sources of oxidative stress and potentially forming toxic lipid compounds when combined with undecomposed lipids (Singh et al. 2017). Fatty acids yield more ATP compared to glycolysis but also consume more oxygen.

Upon cellular stimulation, phospholipase A2 releases precursor substances for various lipid signaling molecules, such as arachidonic acid, through the cell membrane (García-Olmos et al. 2013). These precursors are then converted into prostaglandins and leukotrienes through the cyclooxygenase and lipoxygenase pathways, which are closely associated with exacerbating lung inflammation, with leukotriene B4 being a potential lipid mediator of inflammation (Tanrikulu et al. 2011).

Phosphatidylcholine, the most abundant phospholipid in the lungs, is synthesized from basic substances like glycerol, fatty acids, phosphate, choline, and serine (Zhou et al. 2024). It can be broken down by various phospholipases in the body. Special phosphatidylcholine can form the surfactant of alveolar epithelial cell type 2 (AEC2) (Aydindogan et al. 2019). The phosphatidylcholine pathway plays a significant role in oxidative stress and inflammatory responses. Additionally, lipids play a crucial role in signal transduction (Paliogiannis et al. 2018).

Mitochondrial fatty acid synthesis generates fatty acids through acyl-ACP. Although mitochondrial fatty acid synthesis produces far fewer fatty acids than cytoplasmic fatty acid synthesis and does not replenish cell fatty acids and phospholipids, it may be involved in cell signaling and lipid regulation (Morissette et al. 2015). Acyl-ACP is essential for mitochondrial fatty acid synthesis, mostly present as a soluble form in the mitochondrial matrix, some of which constitute electron transport chain complex I. When mitochondrial fatty acid synthesis is compromised, complexes I, II, and IV are also affected, subsequently influencing the synthesis of longer acyl chains on ACP (Nambiar et al. 2021).

### Lipid regulatory factors in COPD

The mechanisms that control lipid synthesis at the transcriptional level involve several transcription factors, such as Peroxisome Proliferator-Activated Receptors (PPARs), Liver X Receptor (LXR), and SREBP (Sterol Regulatory Element-Binding Protein) (Kilk et al. 2018). While the transcriptional networks regulating lipid homeostasis in other cell types, such as liver and adipocytes, have been extensively studied, limited knowledge exists regarding the transcriptional regulation of lipid homeostasis in the respiratory system. If the potential roles of these transcription factors in COPD are well described, They might have significance in the pathophysiology or therapy of COPD (Wu et al. 2020).

PPAR-γ belongs to the nuclear hormone receptor PPAR subfamily. As a transcription factor, it governs glucose and lipid metabolism, inflammation, and cell proliferation. PPAR-γ pairs with the retinoid X receptor to form a heterodimer, which binds to PPAR response elements and controls the transcription of target genes (Mohamed et al. 2019). In COPD patients, the expression of PPAR-γ is reduced in lung tissues, epithelial cells, and dendritic cells (Zhang et al. 2016). Mice lacking PPAR specifically in epithelial cells show greater vulnerability to chronic cigarette smoke-induced emphysema, along with excessive macrophage accumulation. Selective removal of PPAR-γ in antigen-presenting cells results in spontaneous lung inflammation and emphysema, similar to mice exposed to cigarette smoke (Yasuda et al. 2005). Treating epithelial cells with synthetic (rosiglitazone) or natural PPAR-γ agonists notably increases the expression and activity of PPAR-γ, inhibiting the production and secretion of inflammation cytokines induced by cigarette smoke extract (Yasuda et al. 2005). Follow-up research has demonstrated that PPAR-γ ligands can decrease the production of pro-inflammatory cytokines in COPD alveolar macrophages, such as tumor necrosis factor-alpha and CCL5. Rosiglitazone, a PPAR-γ agonist, can boost the levels of M2 genes associated with anti-inflammatory effects and tissue repair, improving the clearance of apoptotic neutrophils (Yuan et al. 2014). Furthermore, unlike dexamethasone, rosiglitazone decreases neutrophil counts in the airways in a corticosteroid-resistant mouse model of lung inflammation without raising the lung bacterial load. These findings indicate that local activation of PPAR-γ in the airways might serve as an effective therapeutic approach for COPD (Kotlyarov and Bulgakov 2021; Kotlyarov and Kotlyarova 2021).

LXR functions as a detector for cellular cholesterol levels, controlling the transcription of genes related to cholesterol efflux and the degradation of low-density lipoprotein receptors. LXR levels are notably higher in lung tissue extracts from individuals with COPD and smokers, with heightened expression noted in small airways and alveolar epithelium (Sahin et al. 2001). SREBPs act as transcription factors that govern the expression of enzyme genes necessary for cholesterol and unsaturated fatty acid synthesis (Bermudez et al. 2018). Secondhand smoke stimulates liver lipid accumulation by modulating AMPK and SREBP1, suggesting a role for SREBPs in pulmonary diseases that warrants further investigation (Ismail et al. 2015; Chernetska et al. 2020).

### Application of metabolomics techniques in COPD research

Metabolomics is the study of metabolites and their branching pathways of production. Metabolites, as intermediate or final products of biological processes, serve as clues to recent metabolic events (Zeng et al. 2013). Generally, they consist of small molecules (molecular weight < 1500 Da) including amino acids, peptides, nucleic acid components, glycosides, and lipids. When the body is in a pathological or physiological state, changes occur in the nature and quantity of metabolic products within cells. Metabolomics techniques can detect subtle changes in biological pathways by identifying altered metabolites, signaling pathways, and/or biomarkers to help identify new disease phenotypes and gain a deeper understanding of various physiological conditions and abnormal processes (including diseases). This approach may help elucidate the mechanisms of COPD and delay its onset and progression (Koechlin et al. 2004).

**Fig. 3:**
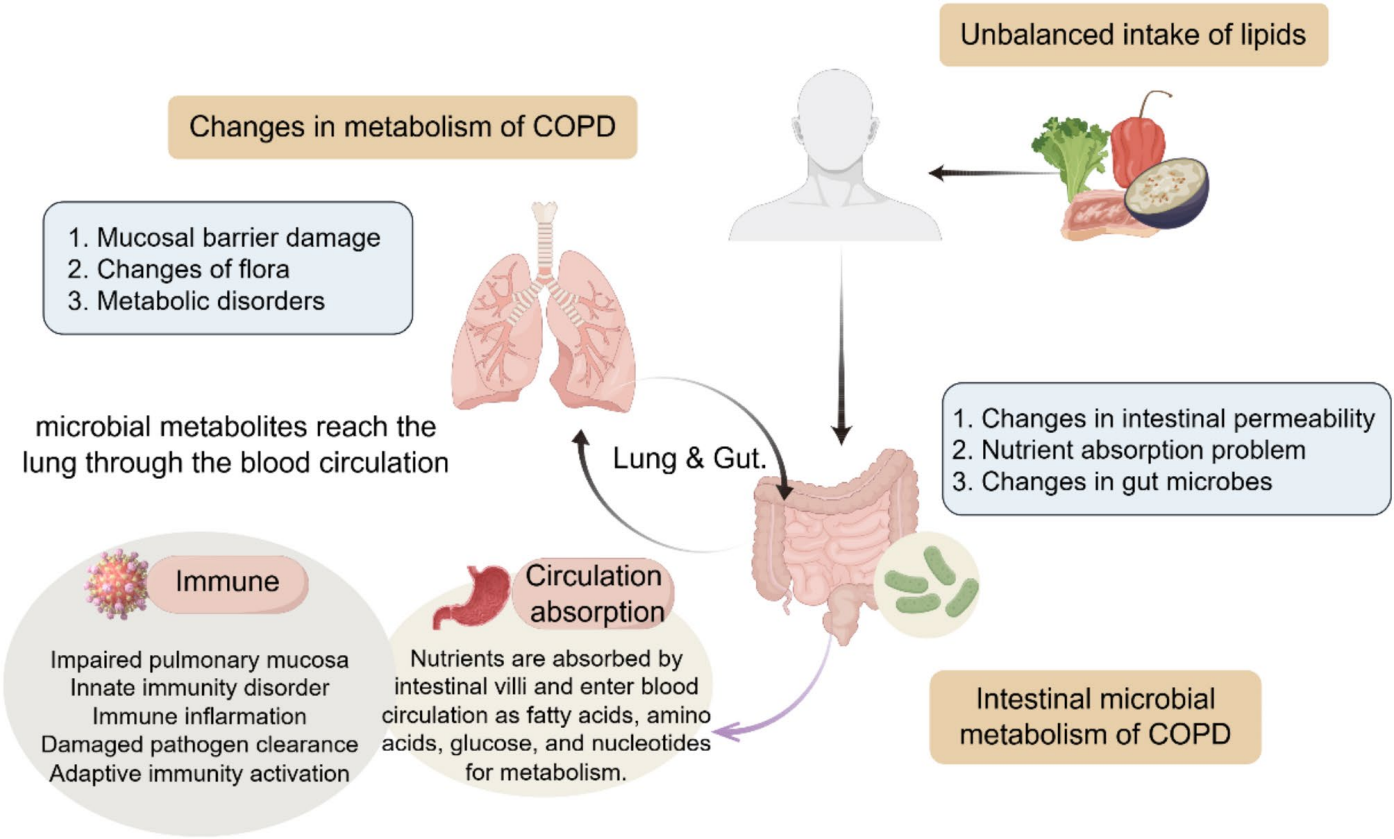
Changes in metabolism of COPD. Metabolic alterations in COPD patients are influenced by an imbalance in lipid intake, impacting both lung tissue and the immune system. The circulation of microbial metabolites from the gut to the lungs leads to changes in both lung and intestinal tissues

The recognized pathogenic mechanisms of COPD include inflammatory mechanisms, protease-antiprotease imbalance, oxidative stress mechanism, and more (Zhao et al. 2018). The combined effects of these mechanisms in COPD result in the secretion of inflammatory mediators, reduced antioxidants, degradation of elastin, and fibroblast generation, resulting in airway remodeling, irreversible airflow limitation, and a series of symptoms (Fig. 3). It has been found that various metabolic pathways and biomolecules are implicated in the progression of COPD, including amino acid metabolism dysfunction, energy metabolism dysfunction, lipid metabolism dysfunction, and oxidative and antioxidant imbalance, via the stimulation of the NF-κB signaling pathway and the emission of inflammatory cytokines, causing protein malnutrition and oxidative stress, which in turn add to the development and worsening of COPD (Corradi et al. 2003).

The regulation of the same lipid molecules may differ among various cells in COPD and could have diverse effects on the ultimate pathogenesis of COPD. Therefore, studying changes in lipid molecules and the metabolites produced from their breakdown in COPD is crucial (Schiffelers et al. 2001). Lipids, also known as fats, mainly include triglycerides, phospholipids, sterols, and other molecular substances. Lipid metabolism abnormalities are observed in COPD, manifested in part by the activation of fatty acid synthesis in pro-inflammatory M1 macrophages (Goyal et al. 2025). Fatty acid synthesis in macrophages primarily relies on glycolysis, the tricarboxylic acid cycle, and the PPP to provide precursors and NADPH. On the other hand, abnormalities are also seen in fatty acid metabolic pathways. Numerous studies have confirmed the close association between COPD and arachidonic acid metabolism and phospholipid metabolism. Arachidonic acid, a highly effective biological signaling molecule and precursor to numerous lipids signaling molecules, is primarily released from membrane phospholipids (Casella et al. 2006). Increased concentrations of its metabolites, such as leukotrienes, thromboxanes, and prostaglandins, are correlated with the severity of COPD. Intracellular arachidonic acid produces leukotrienes under inflammatory stimuli through the lipoxygenase pathway, known for its inflammatory properties. During acute exacerbations of COPD, leukotrienes induce tracheal mucus secretion, increased sputum concentration, inflammation cell infiltration, increased vascular permeability, tissue edema, severe bronchoconstriction, exacerbating airflow limitation (Lu et al. 2018).

Phospholipids, present in cell membranes, are associated with various cellular functions including growth, differentiation, and apoptosis. Phospholipid metabolism is disrupted in COPD. Ceramide, an intermediate product of phospholipids, acts as a second messenger in cell apoptosis and promotes the development of COPD in various ways. Under conditions of oxidative stress or smoke-induced stimuli, ceramide is produced de novo through the hydrolysis of membrane phospholipids, leading to its abnormal accumulation in lung tissue (Bukowska et al. 2015). This accumulation damages the endothelial defense system, alters membrane permeability, induces the formation of membrane channels, leading to the release of pro-apoptotic proteins from mitochondria. This induces apoptosis in alveolar epithelial cells, promotes inflammatory responses, causes macrophage dysfunction, ultimately resulting in bronchial and alveolar damage, and lung disease. Studies have shown that smoke exposure alters phospholipid rheology, leading to increased accumulation of ceramide in membranes and cells, triggering the pathogenesis of COPD-emphysema (Duffney et al. 2018). Additionally, it has been indicated that the activity of ceramide in human serum is negatively associated with a decrease in lung function, exacerbating the progressive deterioration of COPD.

Lung tissue represents an immediate, efficient, and precise method for assessing the metabolic profile of the lungs in COPD patients (Table 1). In a non-targeted metabolomics analysis focusing on male COPD patients’ lung tissue, perturbations in glycerophospholipid metabolism and the biosynthesis pathways of valine, leucine, and isoleucine were identified as significantly associated with male COPD patients (Kaczmarek et al. 2010). Two overlapping metabolites that include phytosphingosine and L-tryptophan were identified as having diagnostic value for early male COPD patients. Furthermore, in a study on the treatment of acute exacerbation of chronic obstructive pulmonary disease (AECOPD) rats with mineral azurite, metabolomics analysis of lung tissues post-treatment revealed potential differences in 15 metabolites, including sphingosine, as well as significant changes in the metabolic pathways of three metabolites, including arachidonic acid, linoleic acid, and sphingolipid metabolism (Jakobsson et al. 1995). The study also demonstrated that mineral azurite treatment was more effective than aminophylline in treating AECOPD.

**Table 1:** Changes in lipid profiles in COPD

Metabolic pathways of Lipids	Targets	Performance	References
**FAO pathway**	Alveolar type II cells	Subchronic cigarette smoke exposure activated oxidative metabolism, affecting FAO and the biosynthetic pathway of surfactant in lung type II cells.	(Agarwal et al. 2014)
	Bronchial epichelial cells	Exposure to cigarette smoke increased FAO, possibly exacerbating the accumulation of ROS, mitochondrial damage, and cell death.	(Jiang et al. 2017)
	Airway smooth muscle cell	In the absence of stimulation, the ability for FAO was reduced.	(Gerasimenko et al. 2017)
**Catabolic lipid mediators**			
Prostanoids	Fibroblasts	Elevated PGE_2_ levels played a role in both airway inflammation and compromised lung healing.	(Nambiar et al. 2021)
Leukotrienes	Sputum	LTB was elevated in induced sputum samples.	(Zhao et al. 2018)
Resolvins	Lung tissue	D-series resolvins in COPD lung samples were altered. Treating with resolving D1 reduced inflammation and boosted macrophage phagocytosis.	(Cakmak et al. 2023)
Nitrated fatty acids	Macrophages	NFA treatment reduced inflammatory cytokine and chemokine production in macrophages.	(Yasuda et al. 2005)
**Fatty acid synthesis pathway transcription factors**	Bronchial epichelial cells	Prolonged cigarette smoke exposure could elevate glucose consumption and boost lipid biosynthesis.	(Jiang et al. 2023)
PPAR-γ	Lung tissue, epithelial cells, and myeloid cells	Downregulation of PPAR-γ in those targets heightened vulnerability to chronic cigarette smoke-induced effects. emphysema and expressior of chemokines.	(Gerasimenko et al. 2017)
LXR	Lung tissue	LXR levels rose in lung tissue, specifically in small airway and alveolar epithelium.	(Higham et al. 2013)
**Cholesterol or cholesterol oxides**	Serum, bronchial epithelial cells	Individuals with very severe COPD exhibited elevated cholesterol levels in their blood. Oxidized cholesterol products were increased in the airways of COPD patients, with hydroxycholesterol playing a role in inflammation and smoke-induced emphysema.	(Zafirova-Ivanovska et al. 2016)
**Phospholipids**	Sputum, Plasma, lung tissue	Sphingolipid levels increased in COPD sputum. Plasma sphingolipids correlated with COPD phenotypes. Elevated ceramide in lung tissue associated with alveolar apoptosis.	(Telenga et al. 2014)

## Reprogramming of lipid metabolism in the treatment of COPD

### Reprogramming of lipid metabolism is involved in the pathogenesis of COPD through immune cells

Immune cells are distributed throughout immune organs and various parts of the body, playing roles in maintaining tissue and organ homeostasis, as well as resisting pathogen invasion. In general, immune cells remain relatively inactive, but they become activated when the body is exposed to factors like infections or injuries, immune cells can rapidly activate and initiate a series of immune responses to preserve the relative equilibrium of body (Guan et al. 2022). During immune cell activation, substantial energy and metabolic intermediates are needed to fulfill biosynthetic requirements, thereby completing proliferation, differentiation, and effector functions (Huang et al. 2024). Different types of immune cells exhibit distinct metabolic pathways during their activation, differentiation, and proliferation processes, which differ significantly from their quiescent states, a phenomenon known as metabolic reprogramming. These changes in metabolic pathways further regulate the phenotype and function of immune cells. Nutrients such as glucose, amino acids, and lipids serve as sources of energy and metabolic intermediates that drive the metabolic changes in immune cells. However, research into how nutrients mediate the activation of different immune subtypes through metabolic reprogramming and confer specific immune functions on immune cells is still not sufficiently comprehensive.

Nutrients and energy metabolism form crucial foundations for maintaining the activity and function of immune cells. On one hand, immune cells degrade glucose, fatty acids, and amino acids to produce ATP to maintain their basic cellular functions and specific immune functions (Nadeem et al. 2005). Glucose, fatty acids, and amino acids can each generate pyruvate, acetyl-CoA, and alpha-ketoglutarate through glycolysis, FAO, and glutamine metabolism pathways, respectively (Ekroos et al. 2020). Acetyl-CoA and alpha-ketoglutarate then enter the mitochondria, where they undergo oxidative phosphorylation through the TCA cycle and the electron transport chain to generate the majority of ATP. In aerobic conditions, pyruvate generated by glycolysis primarily participates in the TCA cycle in the mitochondria and produces a significant amount of ATP through oxidative phosphorylation. In anaerobic or low-oxygen environments, most pyruvate is directly reduced to lactate in the cytosol, generating a small amount of ATP (Couillard et al. 2002). Additionally, activated immune cells such as M1 macrophages, neutrophils, and dendritic cells utilize aerobic glycolysis to rapidly produce ATP to maintain cellular functions. On the other hand, glucose, amino acids, and fatty acids, along with the metabolic intermediates of glycolysis and the TCA cycle, can be uptaken by immune cells directly or as precursors for the biosynthesis of cellular components such as proteins, nucleic acids, and lipids (Koechlin et al. 2005).

Fatty acids can activate the TLR4-NF-κB pathway, resulting in the polarization of M1 macrophages and the release of pro-inflammatory cytokines including monocyte chemoattractant protein-1, TNF-α, and IL-1β. The activation of the NF-κB signaling pathway can also promote the activation of sterol regulatory element binding protein (SREBP), upregulating the expression of downstream enzymes involved in fatty acid synthesis (such as elongases and fatty acid synthase), inducing significant fatty acid synthesis and further promoting the polarization of macrophages towards the M1 phenotype. In contrast, unsaturated fatty acids, particularly polyunsaturated fatty acids, have anti-inflammatory effects (Deja et al. 2014). For instance, docosahexaenoic acid can activate PPARγ and AMPK while inhibiting NF-κB, promoting the polarization of macrophages towards the M2 phenotype. Moreover, conjugated linoleic acid can inhibit the expression of MHC class II, CD80, and CD86 on DCs and their migration to lymph nodes, thereby inhibiting the conversion of initial T cells to Th1 and Th17 phenotypes. Additionally, fatty acid beta-oxidation plays a role in maintaining the balance between Teff and Treg cells, with Teff cells requiring lipid synthesis for membrane construction during rapid growth and proliferation, while Treg cells, which grow relatively slowly with lower biosynthetic demands, mainly rely on FAO for energy.

Short-chain fatty acids (SCFAs) such as acetate, propionate, and butyrate are also involved in energy metabolism and play crucial regulatory roles in the differentiation and function of immune cells (Deja et al. 2014). For macrophages, butyrate can induce metabolic reprogramming towards oxidative phosphorylation and lipid metabolism by upregulating the expression of genes involved in oxidative phosphorylation (such as mitochondrial ATP synthase and NADH dehydrogenase) and lipid metabolism (such as lipoprotein lipase), thereby inducing the polarization of M2 macrophages (Deja et al. 2014; Wong et al. 2022). SCFAs derived from dietary fiber also have effects on altering cell metabolism to promote the effector function of CD8 + T cells. SCFAs bind to the G protein-coupled receptor 41 (GPR41) on the surface of CD8 + T cells, inducing the activation of extracellular signal-regulated kinases (ERK) to enhance glucose uptake and glycolysis, thereby enhancing the effector function of CD8 + T cells. Furthermore, as substrates for FAO, particularly butyrate, SCFAs can promote fatty acid uptake and oxidation to produce acetyl-CoA, enhancing the ability of acetyl-CoA to undergo oxidative phosphorylation through the TCA cycle, a crucial metabolic adaptation induced by butyrate that is necessary for the differentiation into memory CD8 + T cells (Pinho et al. 2007). Upon external stimulation, acetate can be taken up by memory CD8 + T cells and converted to acetyl-CoA, subsequently activating the key enzyme of glycolysis—glyceraldehyde-3-phosphate dehydrogenase through acetylation, enhancing glycolytic capacity, and thereby strengthening the rapid immune recall response of memory CD8 + T cells. SCFAs also significantly affect CD4 + T cells, promoting the differentiation of Th17, Th1, and Treg cells, mainly through the inhibition of histone deacetylase and activation of mTOR. SCFAs also influence the function of other immune cells (Balgoma et al. 2013). For example, SCFAs stimulate glycolysis in B cells, promoting plasma cell differentiation and antibody production. SCFAs can induce the expression of indoleamine 2,3-dioxygenase 1 and aldehyde dehydrogenase in DCs, enhancing their induction of Treg differentiation.

The pro-inflammatory mechanisms of lipid mediators also play an important role in the pathogenesis of COPD. Stimulation of membrane phospholipase A2 leads to the production of arachidonic acid and subsequent thromboxane A2, prostaglandins, and leukotrienes, which may be potential causes of airway inflammation. However, anti-inflammatory lipid mediators, primarily composed of ω-3 fatty acids, include specialized pro-resolving lipid mediators, which consist of five major groups: lipoxins, D-series resolvins, E-series resolvins, protectins, and maresins. Supplementing with SPMs can alleviate lung emphysema by controlling inflammatory responses. Additionally, non-enzymatic reaction products of active nitro and fatty acids, such as nitro-fatty acids, also exhibit anti-inflammatory activity. AMPK can activate the Wnt3α/β-catenin and Nrf2 pathways to reduce lung inflammation and the formation of lung emphysema. Lipid metabolism can stimulate inflammasomes, activate signaling pathways, or induce innate immune responses through oxidative stress and lipid mediators (Kasielski and Nowak 2001). On the other hand, certain lipid metabolism-related substances also have anti-inflammatory effects, but further research is needed to understand the specific transformations and interactions that occur in lipid metabolism-induced pro-inflammatory and anti-inflammatory effects.

### Dietary modulation regulates lipid metabolism

In cases of low dietary fiber intake among COPD subjects, heightened levels of inflammatory cells in peripheral blood relative to normal individuals may instigate alterations in gut microbiota, potentially exacerbating disease progression. Current evidence suggests that a high-fiber diet can mitigate the risk of COPD, with the intake of fiber-rich fruits and vegetables exhibiting protective benefits for lung function (Gencer et al. 2011). Implementing preventive, standardized, and personalized dietary interventions may rectify intestinal dysbiosis in COPD patients, enhancing the production of short-chain fatty acids to curtail inflammation and impede COPD advancement. Adequate intake of specific fatty acids can further ameliorate COPD conditions. Notably, reduced levels of polyunsaturated fatty acids in advanced COPD patients are associated with n-3 polyunsaturated fatty acids inhibiting the TLR4 signaling pathway, with their intake positively correlating with COPD amelioration. Conversely, n-6 polyunsaturated fatty acids tend to stimulate the production of inflammatory mediators (Gerasimenko et al. 2017). Specialized pro-resolving lipid mediators (SPMs), the oxygenated derivatives of these fatty acids, exhibit anti-inflammatory properties by dampening pro-inflammatory factor expression, bolstering anti-inflammatory factor production, and facilitating the shift from pro-inflammatory M1 macrophages to anti-inflammatory M2 macrophages.

### Lipid metabolism reprogramming emerges as a novel treatment strategy for COPD

The Carnitine Palmitoyltransferase (CPT) system, a critical component of FAO, is gaining traction as a therapeutic avenue for a spectrum of mitochondrial FAO-related disorders. Although the precise mechanisms of CPT activators or inhibitors remain elusive, inhibiting CPT1 via etomoxir or silencing the CPT1a gene showcases potential in attenuating oxidative stress levels in cellular models exposed to tobacco smoke, hence enhancing cellular viability (Ignatova et al. 2019). Augmenting L-carnitine levels holds promise in alleviating lung injury and emphysema in COPD patients, alongside enhancing lung functionality in murine COPD models. The FASinhibitor C75 demonstrates the ability to suppress ATP upregulation mediated by fatty acid synthesis, improve mitochondrial function, restore mitochondrial membrane potential, and mitigate cell apoptosis. Further investigations are warranted to evaluate its efficacy in COPD models. FAM13A and the SIRT1 gene collaboratively regulate CPT1a expression, thereby modulating FAO and reactive oxygen species generation, underscoring the potential of gene regulation as a therapeutic avenue for COPD. The anti-inflammatory effects of AMPK pathway activation underscore the need for in-depth research to explore the therapeutic potential of AMPK activators in COPD treatment (Duffney et al. 2018). Combination therapy integrating dasatinib and quercetin exhibits promise in regulating lipid metabolism, mitigating senescent cell burden, and curbing inflammatory cytokine secretion. Low-dose rapamycin, by inhibiting mTOR activity, reduces the expression of pro-inflammatory proteins and stifles mTOR-driven aging (Mizumura and Gon 2021). Inhibition of phospholipase A2 could hold therapeutic potential by curbing the production of pro-inflammatory substances. Pertinently, severe COPD patients often manifest elevated blood cholesterol, oxidized cholesterol products, and altered serum lipoprotein levels, culminating in oxidative stress, inflammation, emphysema progression, lipid accumulation, and compromised cardiovascular function, thereby prompting consideration of statin therapy for COPD patients with concomitant cardiovascular complications.

Membrane transporters facilitating the conveyance of diverse lipid metabolism-related entities represent promising targets for COPD intervention (Wang et al. 2023a, b, c). Noteworthy examples include the organic cation transporter 2, pivotal in L-carnitine transport, offering a viable conduit for the transportation of drugs or drug-loaded nanoparticles bound to carnitine. Fatty Acid Transport Protein 2, implicated in arachidonic acid uptake and prostaglandin E2 activation linked to tumor progression, presents a promising avenue for investigating the downregulation of this transporter as a means to potentially decelerate COPD progression (Zhou et al. 2024). Fatty acids, through Fatty Acid Transport Protein 5 activation, engage Peroxisome Proliferator-Activated Receptors (PPARs) in various cellular functions, with PPARγ, known for its anti-inflammatory properties. Following diminished Fatty Acid Transport Protein 5 expression, a corresponding reduction in PPARγ expression suggests a potential therapeutic target worth exploring in the context of COPD management.

## Conclusion and challenge

Proper lipid metabolism is crucial for maintaining healthy lung function, while disruptions in lipid metabolism contribute to the complex pathogenesis of COPD. Lipid microdomains perform various physiological functions, such as assembling and facilitating signaling pathways. Maintaining lipid membrane asymmetry is a complex and understudied mechanism where several pathways of cellular lipid metabolism converge, encompassing synthesis, absorption, export, and storage. The transport of lipids, including cholesterol, serves as a crucial structural element of lipid microdomains, subject to intricate regulation and potential disruption when smoking. Reprogramming of lipid metabolism presents significant potential in the therapeutic landscape of COPD (Guan et al. 2022). By delving deeply into the mechanisms of disrupted lipid metabolism, novel biomarkers and therapeutic targets can be uncovered, laying the groundwork for precision treatments in COPD. Further exploration of the relationship during lipid metabolism and COPD, and how modulation of lipid metabolism can enhance the prognosis of COPD patients, could help regulate lipid metabolism, ameliorate symptoms, and impede disease progression in COPD individuals. Interventions targeting key metabolic pathways such as FAO and synthesis may aid in alleviating oxidative stress and enhancing mitochondrial function, and decreasing apoptosis, thereby exerting a positive impact on COPD treatment. Moreover, novel therapeutic strategies like modulation of the AMPK pathway, combination drug therapies, and innovative drug delivery systems mediated by membrane transporters exhibit promising therapeutic prospects. Through in-depth research and clinical practice, elucidating the mechanisms of lipid metabolism reprogramming in COPD treatment may pave the way for more effective therapeutic interventions for patients.

## Data Availability

No datasets were generated or analysed during the current study.

## References

[CR92] Agarwal AR, Yin F, Cadenas E. Short-term cigarette smoke exposure leads to metabolic alterations in lung alveolar cells. Am J Respir Cell Mol Biol. 2014;51:284–93. 24625219 10.1165/rcmb.2013-0523OC

[CR25] Agudelo CW, Samaha G, Garcia-Arcos I. Alveolar lipids in pulmonary disease. A review. Lipids Health Dis. 2020a;19:122. 32493486 10.1186/s12944-020-01278-8 PMC7268969

[CR52] Agudelo CW, et al. Decreased surfactant lipids correlate with lung function in chronic obstructive pulmonary disease (COPD). PLoS ONE. 2020b;15:e0228279. 32027677 10.1371/journal.pone.0228279 PMC7004328

[CR51] Arezina R, Chen T, Wang D. Conventional, complementary and alternative medicines: mechanistic insights into therapeutic landscape of chronic obstructive pulmonary disease. Int J Chron Obstruct Pulmon Dis. 2023;18:447–57. 37038544 10.2147/COPD.S393540 PMC10082417

[CR65] Aydindogan E, Penque D, Zoidakis J. Systematic review on recent potential biomarkers of chronic obstructive pulmonary disease. Expert Rev Mol Diagn. 2019;19:37–45. 30560707 10.1080/14737159.2018.1559054

[CR105] Balgoma D, Checa A, Sar DG, Snowden S, Wheelock CE. Quantitative metabolic profiling of lipid mediators. Mol Nutr Food Res. 2013;57:1359–77. 23828856 10.1002/mnfr.201200840

[CR30] Baralla A, et al. Plasma proteomic signatures in early chronic obstructive pulmonary disease. Proteom Clin Appl. 2018;12:e1700088. 10.1002/prca.201700088 29412517

[CR37] Barnes PJ. Mediators of chronic obstructive pulmonary disease. Pharmacol Rev. 2004;56:515–48. 15602009 10.1124/pr.56.4.2

[CR6] Barnes PJ. Inflammatory mechanisms in patients with chronic obstructive pulmonary disease. J Allergy Clin Immunol. 2016;138:16–27. 27373322 10.1016/j.jaci.2016.05.011

[CR77] Bermudez G, et al. Association of metabolic syndrome with the severity of airflow obstruction in patients with chronic obstructive pulmonary disease. J ASEAN Fed Endocr Soc. 2018;33:181–7. 33442125 10.15605/jafes.033.02.11 PMC7784159

[CR55] Biswas S, Hwang JW, Kirkham PA, Rahman I. Pharmacological and dietary antioxidant therapies for chronic obstructive pulmonary disease. Curr Med Chem. 2013;20:1496–530. 22963552 10.2174/0929867311320120004

[CR16] Boots AW, Haenen GR, Bast A. Oxidant metabolism in chronic obstructive pulmonary disease. Eur Respir J Suppl. 2003;46:s14–27. 10.1183/09031936.03.00000403a 14621103

[CR12] Bowerman KL, et al. Disease-associated gut Microbiome and metabolome changes in patients with chronic obstructive pulmonary disease. Nat Commun. 2020;11:5886. 33208745 10.1038/s41467-020-19701-0 PMC7676259

[CR88] Bukowska B, et al. Oxidative stress and damage to erythrocytes in patients with chronic obstructive pulmonary disease–changes in ATPase and acetylcholinesterase activity. Biochem Cell Biol. 2015;93:574–80. 26369587 10.1139/bcb-2015-0066

[CR38] Cakmak A, et al. Metabolomic, oxidative, and inflammatory responses to acute exercise in chronic obstructive pulmonary disease. Heart Lung. 2023;59:52–60. 36724589 10.1016/j.hrtlng.2023.01.011

[CR19] Calvier L, Herz J, Hansmann G. Interplay of Low-Density lipoprotein receptors, LRPs, and lipoproteins in pulmonary hypertension. JACC Basic Transl Sci. 2022;7:164–80. 35257044 10.1016/j.jacbts.2021.09.011 PMC8897182

[CR86] Casella M, et al. No evidence of chromosome damage in chronic obstructive pulmonary disease (COPD). Mutagenesis. 2006;21:167–71. 16567348 10.1093/mutage/gel015

[CR58] Catteau M et al. (2021) Response to Electrostimulation Is Impaired in Muscle Cells from Patients with Chronic Obstructive Pulmonary Disease. *Cells* 10. 10.3390/cells10113002 PMC8616440 34831227

[CR1] Chen H, et al. Lipid metabolism in chronic obstructive pulmonary disease. Int J Chron Obstruct Pulmon Dis. 2019;14:1009–18. 31190786 10.2147/COPD.S196210 PMC6524761

[CR79] Chernetska NV, Stupnytska HY, Fediv OI. The role of MDR1 (C3435T) gene polymorphism in patients with chronic obstructive pulmonary disease associated with type 2 diabetes mellitus. J Med Life. 2020;13:349–55. 33072207 10.25122/jml-2020-0139 PMC7550143

[CR40] Chung KF. Inflammatory mediators in chronic obstructive pulmonary disease. Curr Drug Targets Inflamm Allergy. 2005;4:619–25. 17305518 10.2174/156801005774912806

[CR83] Corradi M, et al. Aldehydes in exhaled breath condensate of patients with chronic obstructive pulmonary disease. Am J Respir Crit Care Med. 2003;167:1380–6. 12522029 10.1164/rccm.200210-1253OC

[CR100] Couillard A, Koechlin C, Cristol JP, Varray A, Prefaut C. Evidence of local exercise-induced systemic oxidative stress in chronic obstructive pulmonary disease patients. Eur Respir J. 2002;20:1123–9. 12449164 10.1183/09031936.02.00014302

[CR44] Dailah HG. (2022) Therapeutic Potential of Small Molecules Targeting Oxidative Stress in the Treatment of Chronic Obstructive Pulmonary Disease (COPD): A Comprehensive Review. *Molecules* 27. 10.3390/molecules27175542 PMC9458015 36080309

[CR102] Deja S, et al. Metabolomics provide new insights on lung cancer staging and discrimination from chronic obstructive pulmonary disease. J Pharm Biomed Anal. 2014;100:369–80. 25213261 10.1016/j.jpba.2014.08.020

[CR45] Drozdovszky O, Barta I, Antus B. Sputum eicosanoid profiling in exacerbations of chronic obstructive pulmonary disease. Respiration. 2014;87:408–15. 24714447 10.1159/000358099

[CR89] Duffney PF, et al. Key roles for lipid mediators in the adaptive immune response. J Clin Invest. 2018;128:2724–31. 30108196 10.1172/JCI97951 PMC6025978

[CR99] Ekroos K, et al. Lipid-based biomarkers for CVD, COPD, and aging - A translational perspective. Prog Lipid Res. 2020;78:101030. 32339553 10.1016/j.plipres.2020.101030

[CR20] Fan LC et al. (2023) Alveolar type II epithelial cell FASN maintains lipid homeostasis in experimental COPD. JCI Insight 8. 10.1172/jci.insight.163403 PMC10543729 37606038

[CR7] Fedele P et al. (2024) Immunonutrition, metabolism, and programmed cell death in lung cancer: translating bench to bedside. Biology (Basel) 13. 10.3390/biology13060409 PMC11201027 38927289

[CR54] Gao P, et al. Respiratory virus infections and adenovirus characteristics during acute exacerbation of chronic obstructive pulmonary disease. Technol Health Care. 2024;32:4203–21. 39058463 10.3233/THC-240010 PMC11613029

[CR62] García-Olmos L, et al. Comorbidity in patients with chronic obstructive pulmonary disease in family practice: a cross sectional study. BMC Fam Pract. 2013;14:11. 23324308 10.1186/1471-2296-14-11 PMC3556139

[CR107] Gencer M, et al. Prolidase activity dysregulation and its correlation with oxidative-antioxidative status in chronic obstructive pulmonary disease. J Clin Lab Anal. 2011;25:8–13. 21254236 10.1002/jcla.20347 PMC6647584

[CR93] Gerasimenko ON, Sukhaterina NA, Shpagin IS. [Role of adipocytokines in the integrated assessment of nutritional status of patients with a combination of hypertension and chronic obstructive pulmonary disease]. Vopr Pitan. 2017;86:29–36. 30695609 10.24411/0042-8833-2017-00057

[CR85] Goyal A, Chopra V, Garg K, Sharma S. (2025) Mechanisms coupling the mTOR pathway to chronic obstructive pulmonary disease (COPD) pathogenesis. Cytokine Growth Factor Rev. 10.1016/j.cytogfr.2024.12.005 39799015

[CR97] Guan S, et al. Metabolic reprogramming by adenosine antagonism and implications in non-small cell lung cancer therapy. Neoplasia. 2022;32:100824. 35914370 10.1016/j.neo.2022.100824 PMC9344351

[CR33] He Q, et al. Ultra-processed food consumption, mediating biomarkers, and risk of chronic obstructive pulmonary disease: a prospective cohort study in the UK biobank. Food Funct. 2023;14:8785–96. 37674411 10.1039/d3fo02069j

[CR94] Higham A, et al. The role of the liver X receptor in chronic obstructive pulmonary disease. Respir Res. 2013;14:106. 24118845 10.1186/1465-9921-14-106 PMC3852990

[CR11] Hsieh MH, et al. Surfactant protein D inhibits lipid-laden foamy macrophages and lung inflammation in chronic obstructive pulmonary disease. Cell Mol Immunol. 2023;20:38–50. 36376488 10.1038/s41423-022-00946-2 PMC9794778

[CR13] Huang Q, et al. Metabolic reprogramming in lung cancer and its clinical implication. Front Pharmacol. 2024;15:1516650. 39744129 10.3389/fphar.2024.1516650 PMC11688361

[CR41] Hunninghake DB. Cardiovascular disease in chronic obstructive pulmonary disease. Proc Am Thorac Soc. 2005;2:44–9. 16113468 10.1513/pats.200410-050SF

[CR108] Ignatova GL, Blinova EV, Antonov VN, Grebneva IV. [Analysis of the impact of vaccination of Pneumococcal infection in patients with chronic obstructive pulmonary disease in combination with diabetes]. Ter Arkh. 2019;91:49–54. 32598610 10.26442/00403660.2019.11.000424

[CR78] Ismail M, Hossain MF, Tanu AR, Shekhar HU. (2015) Effect of spirulina intervention on oxidative stress, antioxidant status, and lipid profile in chronic obstructive pulmonary disease patients. *Biomed Res Int* 2015: 486120. 10.1155/2015/486120 PMC4320919 25685791

[CR91] Jakobsson P, Jorfeldt L, von Schenck H. Fat metabolism and its response to infusion of insulin and glucose in patients with advanced chronic obstructive pulmonary disease. Clin Physiol. 1995;15:319–29. 7554766 10.1111/j.1475-097x.1995.tb00522.x

[CR46] Jiang Z, et al. Genetic control of fatty acid β-Oxidation in chronic obstructive pulmonary disease. Am J Respir Cell Mol Biol. 2017;56:738–48. 28199134 10.1165/rcmb.2016-0282OC PMC5516290

[CR4] Jiang C, Peng M, Dai Z, Chen Q. Screening of lipid Metabolism-Related genes as diagnostic indicators in chronic obstructive pulmonary disease. Int J Chron Obstruct Pulmon Dis. 2023;18:2739–54. 38046983 10.2147/COPD.S428984 PMC10693249

[CR5] Jin C, Yuan P. Implications of lipid droplets in lung cancer: associations with drug resistance. Oncol Lett. 2020;20:2091–104. 32782526 10.3892/ol.2020.11769 PMC7399769

[CR42] Jin F, et al. ARID1A mutations in lung cancer: biology, prognostic role, and therapeutic implications. Trends Mol Med. 2023;29:646–58. 37179132 10.1016/j.molmed.2023.04.005

[CR90] Kaczmarek P, et al. The influence of Simvastatin on selected inflammatory markers in patients with chronic obstructive pulmonary disease. Pol Arch Med Wewn. 2010;120:11–7. 20150839

[CR106] Kasielski M, Nowak D. Long-term administration of N-acetylcysteine decreases hydrogen peroxide exhalation in subjects with chronic obstructive pulmonary disease. Respir Med. 2001;95:448–56. 11421501 10.1053/rmed.2001.1066

[CR23] Kazeminasab S, Emamalizadeh B, Jouyban A, Shoja MM, Khoubnasabjafari M. Macromolecular biomarkers of chronic obstructive pulmonary disease in exhaled breath condensate. Biomark Med. 2020;14:1047–63. 32940079 10.2217/bmm-2020-0121

[CR69] Kilk K et al. (2018) Phenotyping of chronic obstructive pulmonary disease based on the integration of metabolomes and clinical characteristics. Int J Mol Sci 19. 10.3390/ijms19030666 PMC5877527 29495451

[CR81] Koechlin C, et al. Does oxidative stress alter quadriceps endurance in chronic obstructive pulmonary disease? Am J Respir Crit Care Med. 2004;169:1022–7. 15001462 10.1164/rccm.200310-1465OC

[CR101] Koechlin C, et al. Hypoxaemia enhances peripheral muscle oxidative stress in chronic obstructive pulmonary disease. Thorax. 2005;60:834–41. 15964914 10.1136/thx.2004.037531 PMC1747208

[CR26] Kotlyarov S. (2021) Participation of ABCA1 transporter in pathogenesis of chronic obstructive pulmonary disease. Int J Mol Sci 22. 10.3390/ijms22073334 PMC8037621 33805156

[CR48] Kotlyarov S. Analysis of differentially expressed genes and signaling pathways involved in atherosclerosis and chronic obstructive pulmonary disease. Biomol Concepts. 2022;13:34–54. 35189051 10.1515/bmc-2022-0001

[CR9] Kotlyarov S, Bulgakov A. (2021) Lipid Metabolism Disorders in the Comorbid Course of Nonalcoholic Fatty Liver Disease and Chronic Obstructive Pulmonary Disease. *Cells* 10. 10.3390/cells10112978 PMC8616072 34831201

[CR75] Kotlyarov S, Kotlyarova A. (2021) The role of ABC transporters in lipid metabolism and the comorbid course of chronic obstructive pulmonary disease and atherosclerosis. Int J Mol Sci 22. 10.3390/ijms22136711 PMC8269124 34201488

[CR28] Lahousse L, et al. Chronic obstructive pulmonary disease and lipid core carotid artery plaques in the elderly: the Rotterdam study. Am J Respir Crit Care Med. 2013;187:58–64. 23144329 10.1164/rccm.201206-1046OC

[CR43] Li J et al. (2020) Chemerin: A Potential Regulator of Inflammation and Metabolism for Chronic Obstructive Pulmonary Disease and Pulmonary Rehabilitation. *Biomed Res Int* 2020: 4574509. 10.1155/2020/4574509 PMC7166297 32337250

[CR2] Liu G, Summer R. Cellular metabolism in lung health and disease. Annu Rev Physiol. 2019;81:403–28. 30485759 10.1146/annurev-physiol-020518-114640 PMC6853603

[CR14] Liu X, et al. Dihydroquercetin suppresses cigarette smoke induced ferroptosis in the pathogenesis of chronic obstructive pulmonary disease by activating Nrf2-mediated pathway. Phytomedicine. 2022a;96:153894. 34942457 10.1016/j.phymed.2021.153894

[CR47] Liu X, et al. High-coverage lipidomics analysis reveals biomarkers for diagnosis of acute exacerbation of chronic obstructive pulmonary disease. J Chromatogr B Analyt Technol Biomed Life Sci. 2022b;1201–1202:123278. 35561641 10.1016/j.jchromb.2022.123278

[CR87] Lu MC, et al. Effect of oligomeric Proanthocyanidin on the antioxidant status and lung function of patients with chronic obstructive pulmonary disease. Vivo. 2018;32:753–8. 10.21873/invivo.112304 PMC6117753 29936455

[CR27] Lundström SL, et al. Lipid mediator profiling in pulmonary disease. Curr Pharm Biotechnol. 2011;12:1026–52. 21466458 10.2174/138920111795909087

[CR36] Minagawa S, et al. Regulated necrosis in pulmonary disease. A focus on necroptosis and ferroptosis. Am J Respir Cell Mol Biol. 2020;62:554–62. 32017592 10.1165/rcmb.2019-0337TR

[CR57] Mizumura K, Gon Y. (2021) Iron-Regulated reactive oxygen species production and programmed cell death in chronic obstructive pulmonary disease. Antioxid (Basel) 10. 10.3390/antiox10101569 PMC8533398 34679704

[CR71] Mohamed A, Kunda NK, Ross K, Hutcheon GA, Saleem IY. Polymeric nanoparticles for the delivery of MiRNA to treat chronic obstructive pulmonary disease (COPD). Eur J Pharm Biopharm. 2019;136:1–8. 30615927 10.1016/j.ejpb.2019.01.002

[CR67] Morissette MC, Shen P, Thayaparan D, Stämpfli MR. Disruption of pulmonary lipid homeostasis drives cigarette smoke-induced lung inflammation in mice. Eur Respir J. 2015;46:1451–60. 26113683 10.1183/09031936.00216914

[CR98] Nadeem A, Raj HG, Chhabra SK. Increased oxidative stress and altered levels of antioxidants in chronic obstructive pulmonary disease. Inflammation. 2005;29:23–32. 16502343 10.1007/s10753-006-8965-3

[CR50] Nambiar S, Bong How S, Gummer J, Trengove R, Moodley Y. Metabolomics in chronic lung diseases. Respirology. 2020;25:139–48. 30907495 10.1111/resp.13530

[CR68] Nambiar S, et al. Untargeted metabolomics of human plasma reveal lipid markers unique to chronic obstructive pulmonary disease and idiopathic pulmonary fibrosis. Proteom Clin Appl. 2021;15:e2000039. 10.1002/prca.202000039 33580915

[CR66] Paliogiannis P, et al. Circulating malondialdehyde concentrations in patients with stable chronic obstructive pulmonary disease: a systematic review and meta-analysis. Biomark Med. 2018;12:771–81. 29865860 10.2217/bmm-2017-0420

[CR104] Pinho RA, et al. Oxidative stress in chronic obstructive pulmonary disease patients submitted to a rehabilitation program. Respir Med. 2007;101:1830–5. 17376663 10.1016/j.rmed.2007.02.004

[CR49] Pniewska E, Pawliczak R. (2013) The involvement of phospholipases A2 in asthma and chronic obstructive pulmonary disease. *Mediators Inflamm* 2013: 793505. 10.1155/2013/793505 PMC3780701 24089590

[CR53] Rahman I. Oxidative stress in pathogenesis of chronic obstructive pulmonary disease: cellular and molecular mechanisms. Cell Biochem Biophys. 2005;43:167–88. 16043892 10.1385/CBB:43:1:167

[CR76] Sahin U, et al. Lipid peroxidation and glutathione peroxidase activity in chronic obstructive pulmonary disease exacerbation: prognostic value of malondialdehyde. J Basic Clin Physiol Pharmacol. 2001;12:59–68. 11414508 10.1515/jbcpp.2001.12.1.59

[CR39] Santus P, et al. Lipid peroxidation and 5-lipoxygenase activity in chronic obstructive pulmonary disease. Am J Respir Crit Care Med. 2005;171:838–43. 15579728 10.1164/rccm.200404-558OC

[CR84] Schiffelers SL, et al. beta-Adrenoceptor-mediated thermogenesis and lipolysis in patients with chronic obstructive pulmonary disease. Am J Physiol Endocrinol Metab. 2001;280:E357–364. 11158941 10.1152/ajpendo.2001.280.2.E357

[CR60] Sicinska P, et al. Decreased activity of butyrylcholinesterase in blood plasma of patients with chronic obstructive pulmonary disease. Arch Med Sci. 2017;13:645–51. 28507582 10.5114/aoms.2016.60760 PMC5420625

[CR61] Singh S, et al. Evaluation of oxidative stress and antioxidant status in chronic obstructive pulmonary disease. Scand J Immunol. 2017;85:130–7. 28256060 10.1111/sji.12498

[CR34] Singh B, et al. Effect of doxycyline in chronic obstructive pulmonary disease - An exploratory study. Pulm Pharmacol Ther. 2019;58:101831. 31349003 10.1016/j.pupt.2019.101831

[CR35] Takeda Y, Suzuki M, Jin Y, Tachibana I. Preventive role of tetraspanin CD9 in systemic inflammation of chronic obstructive pulmonary disease. Am J Respir Cell Mol Biol. 2015;53:751–60. 26378766 10.1165/rcmb.2015-0122TR

[CR63] Tanrikulu AC, Abakay A, Evliyaoglu O, Palanci Y. Coenzyme Q10, copper, zinc, and lipid peroxidation levels in serum of patients with chronic obstructive pulmonary disease. Biol Trace Elem Res. 2011;143:659–67. 21080098 10.1007/s12011-010-8897-5

[CR96] Telenga ED, et al. Untargeted lipidomic analysis in chronic obstructive pulmonary disease. Uncovering sphingolipids. Am J Respir Crit Care Med. 2014;190:155–64. 24871890 10.1164/rccm.201312-2210OC

[CR32] Viglino D et al. (2017) Nonalcoholic fatty liver disease in chronic obstructive pulmonary disease. Eur Respir J 49. 10.1183/13993003.01923-2016 28596431

[CR18] Wang Y, et al. Differences in the lipid metabolism profile and clinical characteristics between eosinophilic and non-eosinophilic acute exacerbation of chronic obstructive pulmonary disease. Front Mol Biosci. 2023a;10:1204985. 37503537 10.3389/fmolb.2023.1204985 PMC10369057

[CR24] Wang W, et al. The role of Glucagon-Like Peptide-1 receptor agonists in chronic obstructive pulmonary disease. Int J Chron Obstruct Pulmon Dis. 2023b;18:129–37. 36815056 10.2147/COPD.S393323 PMC9939668

[CR31] Wang Q, et al. Ferroptosis, pyroptosis, and Cuproptosis in Alzheimer’s disease. ACS Chem Neurosci. 2023c;14:3564–87. 37703318 10.1021/acschemneuro.3c00343

[CR17] Wang L, et al. Cardiometabolic index and chronic obstructive pulmonary disease: A population-based cross-sectional study. Heart Lung. 2024;68:342–9. 39244841 10.1016/j.hrtlng.2024.09.002

[CR103] Wong BH, et al. The lipid transporter Mfsd2a maintains pulmonary surfactant homeostasis. J Biol Chem. 2022;298:101709. 35150739 10.1016/j.jbc.2022.101709 PMC8914330

[CR70] Wu Y, et al. The epigenetic regulators and metabolic changes in ferroptosis-associated cancer progression. Mol Cancer. 2020;19:39. 32103754 10.1186/s12943-020-01157-x PMC7045519

[CR10] Xu W, et al. Role of ferroptosis in lung diseases. J Inflamm Res. 2021;14:2079–90. 34045882 10.2147/JIR.S307081 PMC8144020

[CR56] Yang L, et al. Circulating metabolomics revealed novel associations between multiple ambient air pollutants exposure and chronic obstructive pulmonary disease incidence: evidence from a prospective cohort study. Environ Pollut. 2024;359:124727. 39147227 10.1016/j.envpol.2024.124727

[CR73] Yasuda H, et al. Increased arterial carboxyhemoglobin concentrations in chronic obstructive pulmonary disease. Am J Respir Crit Care Med. 2005;171:1246–51. 15764730 10.1164/rccm.200407-914OC

[CR21] Yoshida M, et al. Involvement of cigarette smoke-induced epithelial cell ferroptosis in COPD pathogenesis. Nat Commun. 2019;10:3145. 31316058 10.1038/s41467-019-10991-7 PMC6637122

[CR59] Yu S, et al. Recent progress of ferroptosis in lung diseases. Front Cell Dev Biol. 2021;9:789517. 34869391 10.3389/fcell.2021.789517 PMC8635032

[CR74] Yuan Y, et al. Genetic variations in RORα are associated with chronic obstructive pulmonary disease. J Hum Genet. 2014;59:430–6. 24943193 10.1038/jhg.2014.48

[CR95] Zafirova-Ivanovska B, et al. The level of cholesterol in COPD patients with severe and very severe stage of the disease. Open Access Maced J Med Sci. 2016;4:277–82. 27335600 10.3889/oamjms.2016.063 PMC4908745

[CR80] Zeng M, et al. Local and systemic oxidative stress status in chronic obstructive pulmonary disease patients. Can Respir J. 2013;20:35–41. 23457673 10.1155/2013/985382 PMC3628645

[CR72] Zhang X, et al. Gender difference in plasma fatty-acid-binding protein 4 levels in patients with chronic obstructive pulmonary disease. Biosci Rep. 2016;36:e00302. 26823558 10.1042/BSR20150281 PMC4770303

[CR3] Zhang W, et al. GFPT2-Expressing Cancer-Associated fibroblasts mediate metabolic reprogramming in human lung adenocarcinoma. Cancer Res. 2018;78:3445–57. 29760045 10.1158/0008-5472.CAN-17-2928 PMC6030462

[CR82] Zhao P, et al. Integration of transcriptomics, proteomics, metabolomics and systems Pharmacology data to reveal the therapeutic mechanism underlying Chinese herbal bufei Yishen formula for the treatment of chronic obstructive pulmonary disease. Mol Med Rep. 2018;17:5247–57. 29393428 10.3892/mmr.2018.8480 PMC5865990

[CR15] Zhao H, et al. STIM1 is a metabolic checkpoint regulating the invasion and metastasis of hepatocellular carcinoma. Theranostics. 2020;10:6483–99. 32483465 10.7150/thno.44025 PMC7255033

[CR29] Zhao D, et al. Mechanisms of ferroptosis in Alzheimer’s disease and therapeutic effects of natural plant products: A review. Biomed Pharmacother. 2023;164:114312. 37210894 10.1016/j.biopha.2023.114312

[CR64] Zhou Q, Chen Y, Liang Y, Sun Y. (2024) The Role of Lysophospholipid Metabolites LPC and LPA in the Pathogenesis of Chronic Obstructive Pulmonary Disease. *Metabolites* 14. 10.3390/metabo14060317 PMC11205356 38921452

[CR8] Zhou S et al. (2025) Hypoxia studies in non–small cell lung cancer: pathogenesis and clinical implications (Review). Oncol Rep 53. 10.3892/or.2024.8862 PMC11715622 39749693

[CR22] Zhu Y, Choi D, Somanath PR, Zhang D. (2024) Lipid-Laden Macrophages in Pulmonary Diseases. *Cells* 13. 10.3390/cells13110889 PMC11171561 38891022

